# Antibiotic prescribing in remote versus face-to-face consultations for acute respiratory infections in primary care in England: an observational study using target maximum likelihood estimation

**DOI:** 10.1016/j.eclinm.2023.102245

**Published:** 2023-10-03

**Authors:** Emma Vestesson, Kaat De Corte, Paul Chappell, Elizabeth Crellin, Geraldine M. Clarke

**Affiliations:** a The Health Foundation, London, UK; b University College London Great Ormond Street Institute of Child Health, London, UK; c NHS England, London, UK

**Keywords:** General practice in England, Antibiotics, Telemedicine, TMLE, Causal inference, Acute respiratory infections

## Abstract

**Background:**

The COVID-19 pandemic has led to an ongoing increase in the use of remote consultations in general practice in England. Although the evidence is limited, there are concerns that the increase in remote consultations could lead to more antibiotic prescribing.

**Methods:**

In this cohort study, we used patient-level primary care data from the Clinical Practice Research Datalink to estimate the association between consultation mode (remote versus face-to-face) and antibiotic prescribing in England for acute respiratory infections (ARI) between April 2021 and March 2022. Eligibility criteria were applied at both practice-level and patient-level. 400 practices in England were sampled at random and then 600,000 patients were randomly sampled from the eligible patients (whose sex was recorded). Consultations for acute respiratory infections were identified. All antibiotic prescriptions were included, with the exception of antituberculosis drugs and antileprotic drugs, as identified through chapter 5.1 of the British National Formulary. The CPRD Aurum data was linked to the COVID-19 ONS infection survey by region. All analyses were done at the individual level. Repeated consultations from the same patient within 7 days were grouped together. We used targeted maximum likelihood estimation, a causal machine learning method with adjustment for infection type and patient-level, clinician-level and practice-level factors.

**Findings:**

There were 45,997 ARI consultations (34,555 unique patients) within the study period, of which 28,127 were remote and 17,870 were face-to-face. For children, 48% of consultations were remote and, for adults, 66% were remote. For children, 42% of remote and 43% of face-to-face consultations led to an antibiotic prescription; the equivalent values for adults were 52% and 42%, respectively. After adjustment with TMLE, adults with a remote consultation had 23% (odds ratio [OR] 1.23, 95% CI: 1.18–1.29) higher chance of being prescribed antibiotics than if they had been seen face-to-face. We found no significant association between consultation mode and antibiotic prescribing in children (OR 1.04 95% CI: 0.98–1.11).

**Interpretation:**

The higher rates of antibiotic prescribing in remote consultations for adults are cause for concern. We see no significant difference in antibiotic prescribing between consultation mode for children. These findings should inform antimicrobial stewardship activities for health-care professionals and policy makers. Future research should examine differences in guideline-compliance between remote and face-to-face consultations to understand the factors driving antibiotic prescribing in different consultation modes.

**Funding:**

None.

**Research in context**

**Evidence before this study**

Use of remote consultations in general practice has increased rapidly since the onset of the COVID-19 pandemic. Concerns have been raised that antibiotic prescribing rates may be higher in remote compared with face-to-face consultations. Acute respiratory infection (ARI) is the most common reason for an antibiotic prescription in adults making it one of the most important areas of prescription practice for antibiotic use. Empirical studies investigating the differences in antibiotic prescribing rates between online and remote consultations have produced mixed findings, in general and for ARIs specifically. Recent review-type articles on the topic–including a 2020 qualitative systematic review and a 2021 meta-analytic systematic review—have reported mixed results when comparing online and face-to-face consultations with some showing higher and others lower antibiotic prescribing in remote consultations. Furthermore, many of the studies that were included in the reviews were at risk of bias due to a failure to control for demographic and clinical differences between patients in remote versus face-to-face consultations.

**Added value of this study**

This is the first England-wide study estimating the difference in antibiotic prescribing between consultations modes since the start of the COVID pandemic, where both remote and face-to-face consultations are now commonly used. It is also the first study in this setting to apply TMLE—doubly robust causal machine learning method. We found that an adult was 23% more likely to be prescribed an antibiotic for an ARI in a remote compared with a face-to-face consultation with a general practitioner in England. There was no evidence for a difference in children. Our findings are based on an analysis of a representative sample of almost 46,000 GP consultations for ARIs in general practice in England and control for patient, clinician- and practice-level factors that are associated with both consultation mode and with antibiotic prescribing. As such, our findings are at a smaller risk of bias from unobserved confounding than the previous research examining this issue and, therefore, represent an important contribution to the evidence base.

**Implications of all the available evidence**

This study uses rich patient-level data and robust statistical methods and represents an important contribution to the evidence base on antibiotic prescribing in primary care since the start of the COVID pandemic. Taken together with the existing body of evidence on this topic, our results showing higher prescribing in remote consultations are cause for concern. The factors affecting antibiotic prescribing and the interaction with consultation mode are complex and will require further research to unpick. The existing evidence, including this study, have largely focused on prescribing rates, and do not investigate the appropriateness of antibiotics prescribing in remote compared to face-to-face consultations. Further investigation is required to explain the discrepancy in antibiotic prescribing between consultation modes. The growing body of evidence in this area has relevance for future antimicrobial stewardship activities and should be used to inform the ongoing development of antibiotic prescribing guidelines for remote consultations.

## Introduction

The development of antibiotic resistance is a global public health issue largely fuelled by antibiotic overprescribing. Most antibiotic prescribing in England happens in general practice: 72.1% of total antibiotic prescribing in 2021,^1^ of which approximately 20% is inappropriate.^2^ This makes general practice an important focus of antibiotic stewardship activities. The digitalisation of general practice was accelerated by the COVID-19 pandemic and has led to an ongoing increase in the use of remote consultations for all ages.^3^ The full extent of the consequences of this rapid shift towards ‘telemedicine’ is still unclear and one area of concern is increased antibiotic prescribing in remote consultations. A survey of general practitioners (GPs) in the UK reported that 67% of GPs thought that telehealth has increased their antibiotic prescribing to either a great extent or some extent which corroborates this concern.^4^ As a GP cannot observe or examine the patient in a remote consultation in the same way as in a face-to-face consultation, it has been hypothesised that GPs might increase antibiotic prescribing to be ‘on the safe side’.^5^

Acute respiratory infections (ARIs) account for the greatest number of antibiotic prescriptions in UK general practice.^6^ Remote consultations for ARIs could have a role in reducing spread of infection but over-prescription is a particular risk with these conditions. This is because they are often self-limiting and/or commonly caused by viruses rather than bacteria,^7^ and because patients with cold or sore throat symptoms often request or even pressure GPs for antibiotics.^8^

There is limited evidence on the differences in ARI antibiotic prescribing between patients seen face-to-face and those seen remotely–in particular, in a post-pandemic setting where close to 50% of consultations are remote. Some observational studies have shown increases in prescribing in remote consultations for certain ARIs^9^^,^^10^ but other analyses have found a decrease in prescribing in remote consultations^9^^,^^11^ or no difference.^10^^,^^12^ Additionally, a 2021 meta-analytic systematic review^13^ and a 2020 qualitative systematic review^14^ of the impact of remote consultation on antibiotic prescribing both proved inconclusive. Additionally, the majority of the relevant primary studies^9^^,^^11^^,^^12^^,^^15^ and systematic reviews are at risk of bias due to a failure to control for demographic and clinical differences between patients who are seen remotely and face-to-face. Only one primary study in children used matching to adjust for baseline differences in covariates.^10^ A more comprehensive understanding of differences in antibiotic prescribing in remote versus face-to-face ARI consultations is required, to inform remote consultation and antibiotic stewardship policy going forward.

In this study, we compare antibiotic prescribing in patients that were seen remotely by a GP for an ARI compared to patients that were seen face-to-face or using a mix of face-to-face and remote consultations. Analyses were conducted separately for adults and children under 16.

## Methods

### Study design and data

We performed a cohort study using person-level data from the Clinical Practice Research Datalink (CPRD) Aurum between January 2018 and July 2022. The study protocol was approved by CPRD's Research Data Governance (protocol number: 21_000357).

CPRD Aurum is a database with routinely collected data from primary care practices that uses EMIS Web®. CPRD contains data for over 40 million patients from 1332 practices in England as of May 2022. Patients are broadly representative of the English population based on age, sex, and deprivation. CPRD also linked their data to the 2015 indices of multiple deprivation (IMD) at the patient-level, and to the 2011 urban-rural classification at the practice-level. The data was linked to the COVID-19 ONS infection survey by region (23 September 2022 release). The COVID-19 ONS infection survey was a large-scale, nationally representative survey that was conducted in the United Kingdom between December 2020 to March 2023 and collected data on the prevalence of COVID-19 infection in the community. This was included to control for changes in the prevalence of COVID-19 during the study period.

We selected the 600,000 patients from 400 practices rather than sample from all practices as we wanted to be able to calculate the proportion of remote consultations per practice. By sampling from 400 practices, we expected to get 10–15% of the list size at each practice and therefore the practice-level variables would be more reliable. This enabled us to check for practices with a very high proportion of remote or face-to-face consultations as this would be a sign of poor coding. Cohort eligibility criteria.

We studied patients registered at general practices that participated in CPRD Aurum. Eligibility criteria were applied at both practice- and patient-level. 400 practices in England were sampled at random. Eligible patients were those with acceptable data quality (verified by CPRD); registered at one of the 400 practices at any point between January 2018 and March 2021; recorded as either male or female sex; and eligible for area-level linkage to the index of multiple deprivation (IMD). 600,000 patients were then randomly sampled from the eligible patients. Three GP practices were further identified by CPRD as having duplication issues and were excluded from the analysis.

### Analysis dataset

Consultations for acute respiratory infections were identified (see Supplemental Materials for codelists). This list was based on previously published lists of read codes.^16^ There were five subgroups of ARIs: lower respiratory tract infections (LRTI), upper respiratory tract infections (URTI), sinusitis, otitis externa and otitis media as well as COVID. A consultation could be coded as more than one infection subtype. As it is not possible to determine what the primary diagnosis or main reason for consultation is in CPRD, all recorded diagnosis codes for that consultation were used to identify ARIs.

Antibiotics from chapter 5.1 of the British National Formulary (BNF) with the exclusion of antituberculosis drugs (5.1.9) and antileprotic drugs (5.1.10) were included. Antibiotic prescriptions were linked to a consultation either using the observation and problem IDs provided by CPRD, or if they were issued on the same date as an ARI consultation.

Consultations were classified by mode of delivery as either remote (by telephone, video, SMS, and through the internet) or face-to-face (at the GP surgery or at home) consultations based on information in the consultation table for consultation mode or observations recorded during the consultation. Where a consultation's mode was unclear, it was assumed to be face-to-face. For a more comprehensive description of these classification methods, readers are referred to^3^ where the methodology has been detailed in depth. Only consultations carried out by GPs were included. GPs are likely to be responsible for most of the antibiotic prescribing for ARIs in general practice and have a higher proportion of remote consultations than other health care professionals. There were 67,324 ARI consultations carried out by GPs between 1 April 2021 and 22 March 2022. This period spans the removal of the stay-at-home order to the most recent available data. ARI consultation records for the same patient happening on the same day were grouped together as these were likely to be part of the same consultation (49,451). ARI consultations happening in a 7-day period were grouped together retaining the date of first consultation so that patients were not included multiple times in a short time period. If the consultation mode was the same for all grouped consultations, it was coded as such; if there was a mix of face-to-face and remote consultations, it was recorded as mixed and included in the face-to-face category for the main analysis. If antibiotics were prescribed in any of the grouped consultations, the prescription was retained in the analysis dataset.

No data was imputed for any variables. A very small number of patients (less than 15) that did not have IMD recorded were excluded from the analysis.

### Variable selection

To optimise the potential for variable adjustment as well as to ensure the exchangeability at baseline of the treatment arms, as many characteristics as possible were included in the dataset at several covariate levels: consultation-, patient-, clinician-, and practice-level. We adjusted for factors (or their proxies) known to be associated with antibiotic prescribing. These included comorbidities such as asthma or COPD,^17^, ^18^, ^19^, ^20^ infection type, health need, rurality, ethnicity, deprivation,^21^ region,^22^ CCG,^23^ clinician job role at practice,^20^^,^^24^ and overall practice consultation rates based on all patients in the sample.

We also adjusted for variables that were identified through conversations with practising GPs to be associated with either antibiotic prescribing or having a remote consultation. The selection of GPs and the identification of additional variables was not systematic. These included COVID-19 infection in the last 7, 30 or 365 days before the consultation, number of remote or face-to-face consultations in the last 7, 30 or 365 days before the consultation and regional ONS COVID-19 infection prevalence.

All covariates were included in both the treatment and outcome models. See Table S2 for a table of covariates that were included for adults and children.

### Key assumptions for causal identification

To identify the treatment effect of consultation mode on antibiotic prescribing, we made the following causal assumptions. The stable unit treatment value assumption, i.e., the treatment status of any individual did not affect the potential outcomes of other individuals. This likely holds as one patient having a remote consultation is unlikely to have an impact on the decision to prescribe antibiotics to a different patient. The second assumption was no unmeasured confounding. In other words, the exposure mechanism and potential outcomes were independent after conditioning on our defined set of covariates. We have minimised the risk of unmeasured confounding through our inclusion of a large selection of relevant patient-, clinician- and practice-level factors that could be linked to both exposure (consultation modality) and outcome (antibiotic prescribing). Thirdly, we assumed positivity, i.e., within strata of the set of covariates, all consultations had a nonzero probability of receiving either exposure condition. By design, only patients with a respiratory infection were included and those can be seen both remotely or face-to-face unlike consultations for other purposes such as vaccinations or wound management. We checked minimum and maximum of the propensity scores from the treatment model for violations of the positivity assumption.

We defined the statistical target parameters as the average treatment effect (ATE) and the odds ratio (OR).

### Validation of exchangeability assumption

The standardised mean difference (SMD) was used to assess how similar the distribution of covariates was between remote and face-to-face consultations. A 10% difference was considered large enough to be noteworthy. For statistical disclosure reasons, summary statistics were suppressed in groups with fewer than 10 consultations.

### Statistical analysis

The exposure is a binary consultation mode variable (remote codes as 1 and face-to-face/mixed as 0) and the outcome is a binary antibiotic prescription variable (antibiotics prescribed coded as 1/antibiotics not prescribed coded as 0).

We used targeted maximum likelihood estimation (TMLE) to estimate the difference in prescribing rates between patients seen remotely and those seen face-to-face. TMLE is a semi-parametric approach used to estimate causal effects in observational data. TMLE is often referred to as a causal machine learning method as it combines elements of machine learning and causal inference. First it creates initial estimates of the treatment and outcome models. Through the construction of a “clever covariate,” which is a function of the treatment, outcome, and propensity score (the probability of treatment assignment given a set of observed covariates), TMLE refines the initial estimate of the causal effect optimising the bias-variance trade-off and estimating the statistical target parameters of interest, in this study the ATE and OR. This double robustness property of TMLE provides protection against potential model misspecifications, thus offering reliable causal effect estimation. TMLE relies on cross-validation to minimise overfitting. When used in combination with a superlearner, it can handle high-dimensional data, which makes it a powerful tool to estimate causal effects.

We used 10-fold cross-validation. We clustered the cross-validation by GP practice so that all observations from the same practice would be within the same cross-validation fold, accounting for intra-practice correlations. In addition to this, we clustered at the individual level in the relevant models comprising the ensemble model, to account for patients appearing multiple times in the data. To ensure consistent prevalence of the binary outcome across cross-validation folds, stratification was performed based on the outcome variable (see Supplemental Materials S3 for more details).

We included a range of models with different strengths in the superlearner, following best practice guidelines based on our data (see Supplemental Materials S3 for more details). Logistic model; a Generalised Additive Model to allow flexibility in capturing nonlinear relationships without a priori specification of the functional form; Random Forest and XGBoost, both tree-based machine learning methods to capture complex interactions and identify key features; Multivariate Adaptive Regression Splines (MARS) model as it can use basis functions (like splines) to model nonlinear relationships; and a lasso net logistic model helped in feature selection by penalising less important variables and reducing them to zero, thus improving model sparsity. Together, these models formed a comprehensive ensemble capturing a range of potential data structures and relationships. Parameter tuning details can be found in Supplemental Materials S3. Using a discrete super learner—which compares the ensemble model with the individual learners—to pick the final model was not computationally feasible. We used non-negative linear least squares as the metalearner for the ensemble model, which is a proven strategy for optimally combining these diverse learners. As this has been proven to perform at least as well as any individual model asymptotically, we considered this a reasonable simplification. The analysis was carried out using R 4.0.2 using sl3 and tmle3 for the modelling.

We reran the models without the mixed consultations that were included in the face-to-face group in the main analysis.

### Role of the funding source

No external funding was received.

## Results

There were 45,997 consultations for ARIs (34,555 unique patients), of which 61% (28,127) were remote and 39% (17,870) face-to-face. For children, 48% of consultations were remote whereas for adults 66% were remote. Antibiotics were prescribed in 48% of all consultations for adults, and in 52% of remote and 42% of face-to-face consultations. For children, 43% of all consultations led to antibiotic prescriptions with 42% in remote and 43% in face-to-face consultations (Table 1). The median age was 4 in children (0–16) and 49 for adults (16+). In children, 52% of consultations were male compared to only 38% in adults. Ethnicity was missing for a higher proportion of consultations for children than for adults. Adult consultations were evenly distributed across the IMD quintiles but there was a slight over-representation of child consultations in the most deprived IMD quintile (Table 1).

**Table 1:** Baseline characteristics based on consultations as the unit of analysis.

	Adults			Children		
	Face-to-face, N = 10,980	Remote, N = 21,741	Overall, N = 32,721	Face-to-face, N = 6890	Remote, N = 6386	Overall, N = 13,276
GP practices	380	384	384	378	372	382
Unique patients	9697	17,728	25,145	5454	5190	9410
Age (years)	49 (34, 65)	50 (34, 65)	49 (34, 65)	3.8 (2.1, 7.3)	4.4 (2.5, 8.7)	4.1 (2.3, 8.0)
Sex						
Male	4128 (38%)	7820 (36%)	11,948 (37%)	3631 (53%)	3246 (51%)	6877 (52%)
Female	9552 (62%)	13,921 (64%)	20,773 (63%)	3259 (49%)	3150 (49%)	6399 (48%)
Antibiotics prescribed	4602 (42%)	11,231 (52%)	15,833 (48%)	2950 (43%)	2712 (42%)	5662 (43%)
GP registrar	1159 (11%)	1521 (7.0%)	2680 (8.2%)	667 (9.7%)	431 (6.7%)	1098 (8.3%)
General medical practitioner	7419 (68%)	15,437 (71%)	22,856 (70%)	4678 (68%)	4489 (70%)	9167 (69%)
Salaried GP	1676 (15%)	3037 (14%)	4713 (14%)	1071 (16%)	849 (13%)	1920 (14%)
Locum GP	461 (4.2%)	730 (3.4%)	1191 (3.6%)	317 (4.6%)	264 (4.1%)	581 (4.4%)
Sessional GP	503 (4.6%)	995 (4.6%)	1498 (4.6%)	346 (5.0%)	352 (5.5%)	698 (5.3%)
Associate practitioner GP	130 (1.2%)	258 (1.2%)	388 (1.2%)	67 (1.0%)	52 (0.8%)	119 (0.9%)
IMD 2015						
1 - least deprived	2320 (21%)	4373 (20%)	6693 (20%)	1284 (19%)	1120 (18%)	2404 (18%)
2	2253 (21%)	4152 (19%)	6405 (20%)	1312 (19%)	1080 (17%)	2392 (18%)
3	2220 (20%)	4291 (20%)	6511 (20%)	1370 (20%)	1193 (19%)	2563 (19%)
4	2011 (18%)	4262 (20%)	6273 (19%)	1248 (18%)	1383 (22%)	2631 (20%)
5 - most deprived	2176 (20%)	4663 (21%)	6839 (21%)	1676 (24%)	1610 (25%)	3286 (25%)
Ethnic group						
White	8737 (80%)	17,483 (80%)	26,220 (80%)	4221 (61%)	3947 (62%)	8168 (62%)
Mixed	138 (1.3%)	276 (1.3%)	414 (1.3%)	180 (2.6%)	187 (2.9%)	367 (2.8%)
Asian or Asian British	706 (6.4%)	1384 (6.4%)	2090 (6.4%)	517 (7.5%)	559 (8.8%)	1076 (8.1%)
Black	325 (3.0%)	580 (2.7%)	905 (2.8%)	191 (2.8%)	178 (2.8%)	369 (2.8%)
Other ethnic groups	129 (1.2%)	251 (1.2%)	380 (1.2%)	114 (1.7%)	123 (1.9%)	237 (1.8%)
(Missing)	945 (8.6%)	1767 (8.1%)	2712 (8.3%)	1667 (24%)	1392 (22%)	3059 (23%)
Cons in last 7 days	1957 (18%)	2782 (13%)	4739 (14%)	821 (12%)	521 (8.2%)	1342 (10%)
Cons in last 30 days	4725 (43%)	8342 (38%)	13,067 (40%)	2200 (32%)	1687 (26%)	3887 (29%)
Cons in last 365 days	9639 (88%)	19,104 (88%)	28,743 (88%)	5529 (80%)	5006 (78%)	10,535 (79%)
Face-to-face cons in last 7 days	845 (7.7%)	1305 (6.0%)	2150 (6.6%)	379 (5.5%)	251 (3.9%)	630 (4.7%)
Face-to-face cons in last 30 days	2760 (25%)	4467 (21%)	7227 (22%)	1338 (19%)	903 (14%)	2241 (17%)
Face-to-face cons in last 365 days	8037 (73%)	15,187 (70%)	23,224 (71%)	4444 (64%)	3664 (57%)	8108 (61%)
Remote cons in last 7 days	1460 (13%)	2059 (9.5%)	3519 (11%)	651 (9.4%)	414 (6.5%)	1065 (8.0%)
Remote cons in last 30 days	3601 (33%)	6917 (32%)	10,518 (32%)	1715 (25%)	1430 (22%)	3145 (24%)
Remote cons in last 365 days	8968 (82%)	18,208 (84%)	27,176 (83%)	5073 (74%)	4772 (75%)	9845 (74%)
Antibiotic in last 7 days	484 (4.4%)	875 (4.0%)	1359 (4.2%)	122 (1.8%)	116 (1.8%)	238 (1.8%)
Antibiotic in last 30 days	1794 (16%)	3302 (15%)	5096 (16%)	700 (10%)	555 (8.7%)	1255 (9.5%)
Antibiotic in last 365 days	4994 (45%)	10,462 (48%)	15,456 (47%)	2749 (40%)	2428 (38%)	5177 (39%)
Other cancer	12 (0.1%)	17 (<0.1%)	29 (<0.1%)	NA	NA	NA
Depression	2607 (24%)	5690 (26%)	8297 (25%)	16 (0.2%)	17 (0.3%)	33 (0.2%)
Dementia	137 (1.2%)	209 (1.0%)	346 (1.1%)	NA	NA	NA
Psychosis	19 (0.2%)	60 (0.3%)	79 (0.2%)	NA	NA	NA
Anxiety	243 (2.2%)	591 (2.7%)	834 (2.5%)	NA	NA	NA
Other respiratory	232 (2.1%)	616 (2.8%)	848 (2.6%)	NA	NA	NA
Other musculoskeletal	1617 (15%)	3239 (15%)	4856 (15%)	NA	NA	NA
Asthma	1665 (15%)	3926 (18%)	5591 (17%)	255 (3.7%)	295 (4.6%)	550 (4.1%)
COPD	478 (4.4%)	1199 (5.5%)	1677 (5.1%)	NA	NA	NA
Skin condition	4918 (45%)	9660 (44%)	14,578 (45%)	2128 (31%)	1972 (31%)	4100 (31%)
Eye condition	931 (8.5%)	1913 (8.8%)	2844 (8.7%)	NA	NA	NA
Hypertension	2496 (23%)	5026 (23%)	7522 (23%)	NA	NA	NA
Other genitourinary	769 (7.0%)	1635 (7.5%)	2404 (7.3%)	12 (0.2%)	28 (0.4%)	40 (0.3%)
Epilepsy	257 (2.3%)	493 (2.3%)	750 (2.3%)	40 (0.6%)	43 (0.7%)	83 (0.6%)
Haemm immun condition	856 (7.8%)	1749 (8.0%)	2605 (8.0%)	49 (0.7%)	67 (1.0%)	116 (0.9%)
Obesity	1345 (12%)	2787 (13%)	4132 (13%)	25 (0.4%)	28 (0.4%)	53 (0.4%)
Neurological condition	1589 (14%)	3112 (14%)	4701 (14%)	32 (0.5%)	42 (0.7%)	74 (0.6%)
Ear condition	646 (5.9%)	1150 (5.3%)	1796 (5.5%)	NA	NA	NA
Chronic liver disease	34 (0.3%)	86 (0.4%)	120 (0.4%)	NA	NA	NA
Other endocrine	50 (0.5%)	83 (0.4%)	133 (0.4%)	NA	NA	NA
Other digestive	86 (0.8%)	193 (0.9%)	279 (0.9%)	NA	NA	NA
Infectious disease (HIV, viral hepatatis)	27 (0.2%)	56 (0.3%)	83 (0.3%)	NA	NA	NA
Other circulatory	33 (0.3%)	83 (0.4%)	116 (0.4%)	NA	NA	NA
ARI cons in last 365 days	3392 (31%)	6898 (32%)	10,290 (31%)	2620 (38%)	2271 (36%)	4891 (37%)
ARI cons in last 30 days	1343 (12%)	2494 (11%)	3837 (12%)	736 (11%)	559 (8.8%)	1295 (9.8%)
ARI cons in last 7 days	259 (2.4%)	442 (2.0%)	701 (2.1%)	82 (1.2%)	67 (1.0%)	149 (1.1%)
ARI remote cons in last 365 days	2668 (24%)	6166 (28%)	8834 (27%)	1939 (28%)	1896 (30%)	3835 (29%)
ARI remote cons in last 30 days	913 (8.3%)	2104 (9.7%)	3017 (9.2%)	424 (6.2%)	412 (6.5%)	836 (6.3%)
ARI remote cons in last 7 days	170 (1.5%)	362 (1.7%)	532 (1.6%)	48 (0.7%)	46 (0.7%)	94 (0.7%)
ARI face-to-face cons in last 365 days	1626 (15%)	2238 (10%)	3864 (12%)	1663 (24%)	1034 (16%)	2697 (20%)
ARI face-to-face cons in last 30 days	596 (5.4%)	731 (3.4%)	1327 (4.1%)	457 (6.6%)	241 (3.8%)	698 (5.3%)
ARI face-to-face cons in last 7 days	102 (0.9%)	112 (0.5%)	214 (0.7%)	46 (0.7%)	29 (0.5%)	75 (0.6%)
COVID-19 infection recorded in last 30 days	2263 (21%)	3962 (18%)	6225 (19%)	609 (8.8%)	430 (6.7%)	1039 (7.8%)
COVID-19 infection recorded in last 7 days	2053 (19%)	3589 (17%)	5642 (17%)	526 (7.6%)	356 (5.6%)	882 (6.6%)
Antibiotics/10k patient days in prior year (practice)	10.47 (8.83, 12.29)	10.57 (8.96, 12.28)	10.55 (8.83, 12.28)	10.43 (8.70, 12.28)	10.47 (8.57, 12.19)	10.47 (8.65, 12.23)
Cons/10k patient days in prior year (practice)	81 (69, 100)	81 (69, 98)	81 (69, 98)	78 (66, 98)	80 (67, 97)	80 (67, 97)
ONS COVID infection rate	1.74 (1.22, 3.76)	1.59 (1.05, 3.27)	1.64 (1.11, 3.52)	1.56 (1.01, 2.22)	1.48 (0.50, 2.04)	1.53 (0.88, 2.11)
Practice list size	12,518 (7977, 17,844)	12,426 (7951, 18,752)	12,478 (7977, 18,697)	12,518 (7951, 18,703)	12,600 (7629, 19,776)	12,518 (7752, 19,161)
Urban practice	8958 (82%)	18,893 (87%)	27,851 (85%)	5933 (86%)	5784 (91%)	11,717 (88%)
Region						
East Midlands	282 (2.6%)	565 (2.6%)	847 (2.6%)	184 (2.7%)	121 (1.9%)	305 (2.3%)
East of England	427 (3.9%)	1142 (5.3%)	1569 (4.8%)	276 (4.0%)	332 (5.2%)	608 (4.6%)
London	2148 (20%)	3927 (18%)	6075 (19%)	1650 (24%)	1327 (21%)	2977 (22%)
North East	241 (2.2%)	497 (2.3%)	738 (2.3%)	150 (2.2%)	97 (1.5%)	247 (1.9%)
North West	2854 (26%)	5085 (23%)	7939 (24%)	1732 (25%)	1421 (22%)	3153 (24%)
South East	1890 (17%)	3554 (16%)	5444 (17%)	1135 (16%)	1102 (17%)	2237 (17%)
South West	1058 (9.6%)	2243 (10%)	3301 (10%)	478 (6.9%)	488 (7.6%)	966 (7.3%)
West Midlands	1559 (14%)	3541 (16%)	5100 (16%)	1047 (15%)	1148 (18%)	2195 (17%)
Yorkshire and the Humber	521 (4.7%)	1187 (5.5%)	1708 (5.2%)	238 (3.5%)	350 (5.5%)	588 (4.4%)

Abbreviation: Cons is short for consultations.

**Fig. 1:**
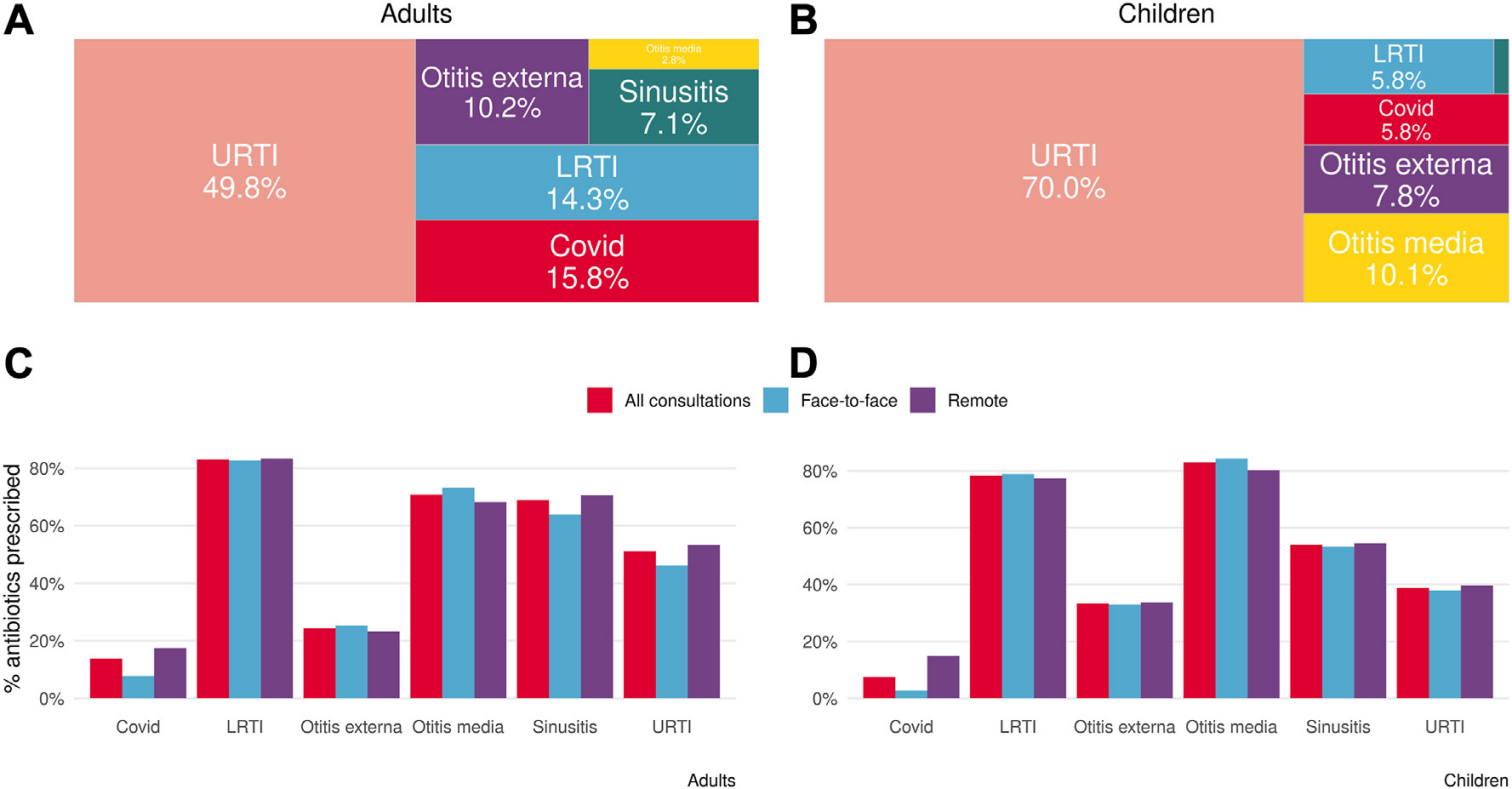
Breakdown of infection type codes (% of total) (A & B) and proportion of consultations leading to an antibiotic prescription by consultation mode and infection.

Most consultations were only associated with one infection type (97.5% adults and 97.4% children). The proportion of consultations only associated with one infection was marginally higher in face-to-face compared to remote consultations for both adults and children (2.9% versus 2.3% for adults and 2.6% versus 3.5% for children). Regardless of the type of infection, adults had a higher proportion of remote consultations compared to children. For a URTI, 71% of adults were seen remotely compared with just 53% of children (Figure S1). The most common ARI type for both adults and children was URTI (49.8% and 70.0% of all diagnoses respectively) but the frequency of some infection types differed substantially between adults and children, notably LRTI being high in adults but low in children (14.3% and 5.8% respectively) and otitis media being the opposite (2.8% in adults and 10.1% in children) (Fig. 1A and B). LRTI and otitis media had the highest antibiotic prescribing rates within the study (LRTI: 82.6% adults and 78.9% children; Otitis media: 73.2% adults and 84.3% children) so the prevalence of these infections will highly influence baseline prescribing rates. Antibiotics were more commonly prescribed for adults with URTIs in remote rather than face-to-face consultations (53.3% compared to 46.1%) whereas there was very little difference in prescribing rates for children (39.6% compared to 37.9%). Antibiotics were more often prescribed in remote consultations for COVID infection for both adults (17.5% versus 7.8%) and children (15.0% versuss 2.7%) (Fig. 1C and D).

**Fig. 2:**
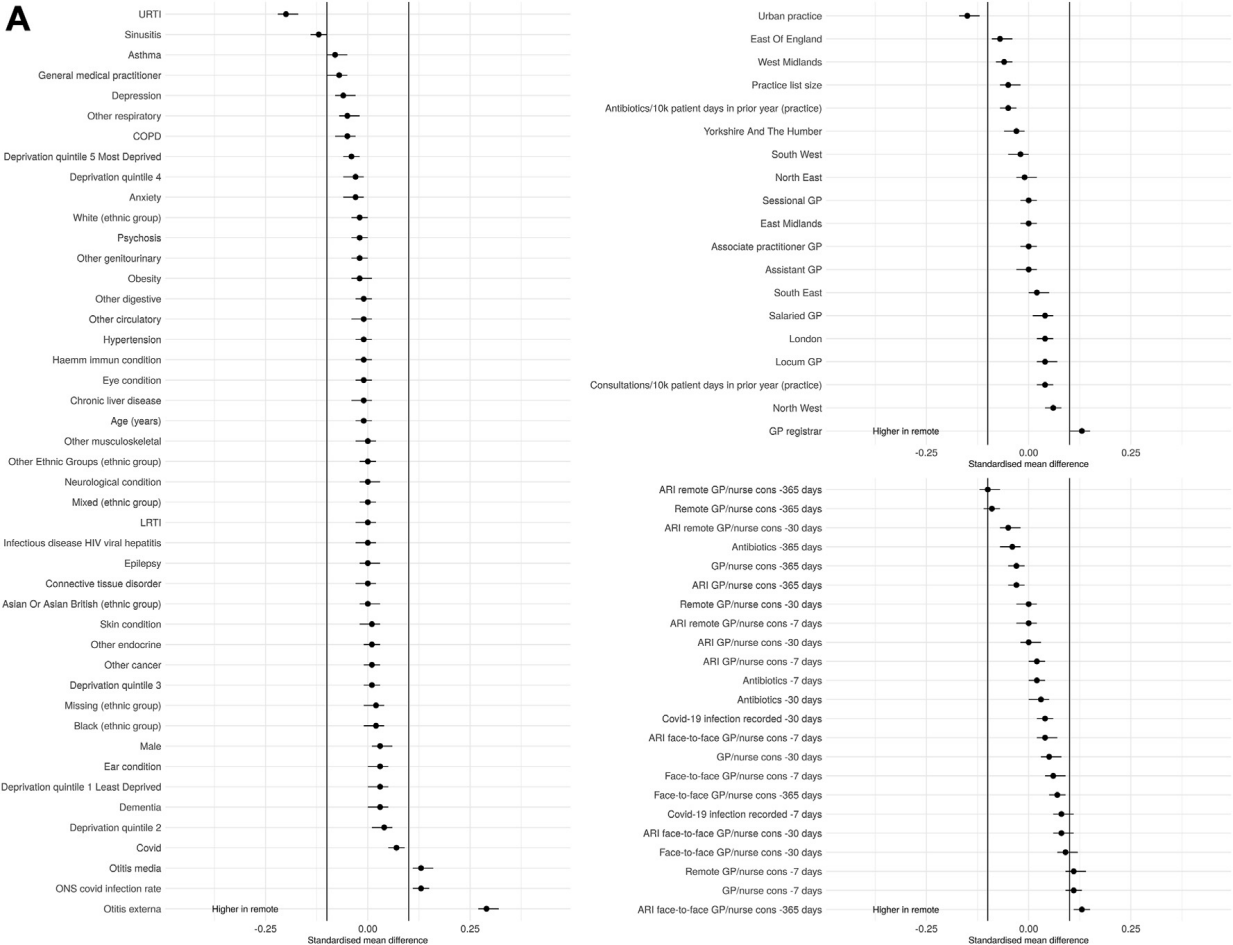

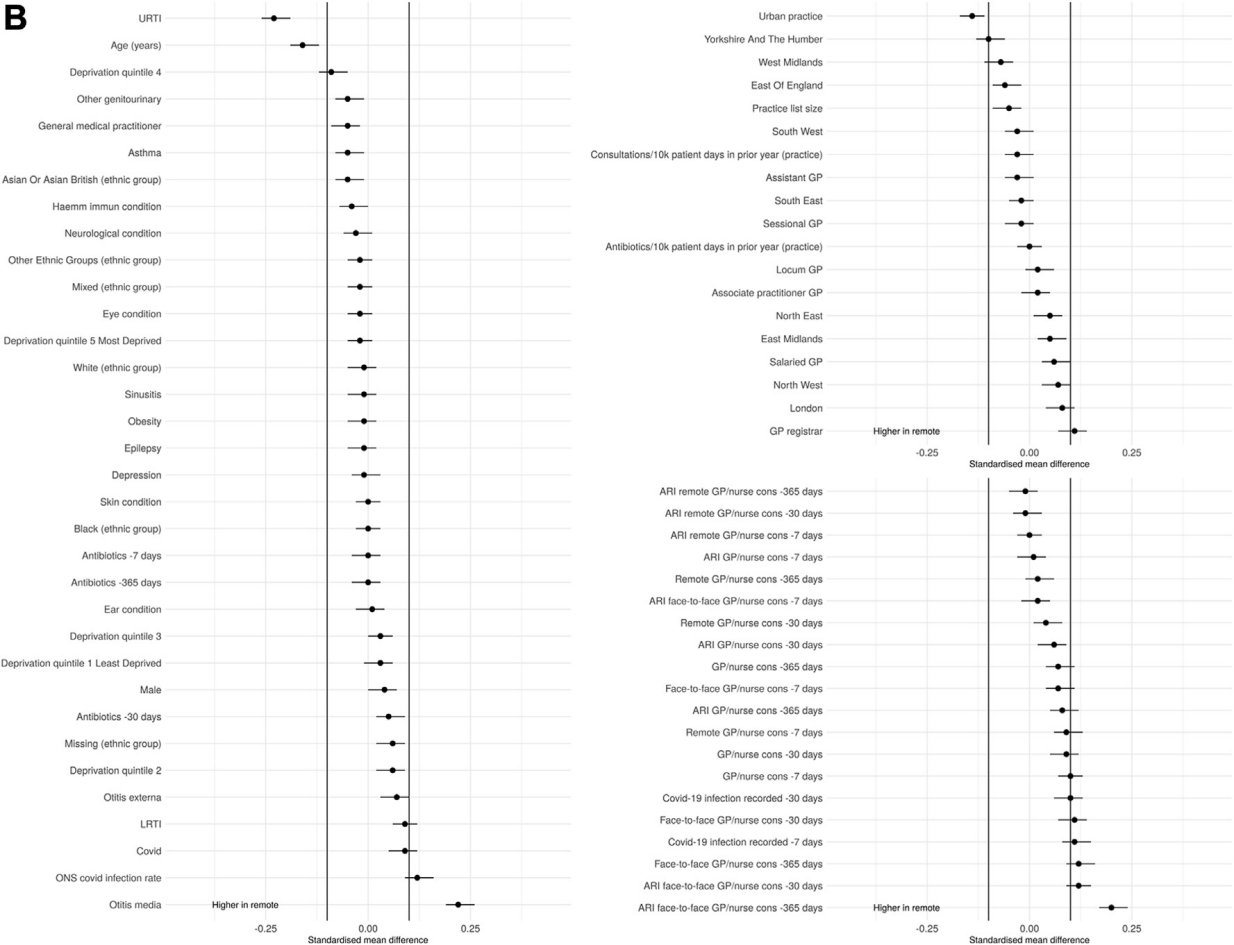
A SMD adults. B SMD children.

There were some differences in the baseline characteristics of patients having remote compared with face-to-face consultations (Table 1, Fig. 2). For both adults and children, consultations were more likely to be remote when related to a URTI or in an urban practice and more likely to be face-to-face when related to an otitis media diagnosis or with a GP registrar. The number of previous ARI face-to-face consultations in the last year was also associated with being seen face-to-face.

The minimum and the maximum propensity scores were 0.037 and 0.944, respectively, for children; and 0.039 and 0.962, respectively, for adults which suggests the positivity assumption is reasonable.

Using TMLE, we estimated that 44.7% (95% CI: 43.7, 45.7) of adults would have been prescribed antibiotics if they had all been seen face-to-face with 49.9% (95% CI: 49.2, 50.2) if seen remotely, which corresponds to a difference in average treatment effect of 5.1% (95% CI: 4.0, 6.2) and an odds ratio of 1.23 (95% CI: 1.18, 1.29).

In children, we estimated that 41.8% (95% CI: 40.6, 43.0) would have been prescribed antibiotics if they had all been seen face-to-face with 42.8% (95% CI: 41.6, 43.9) if seen remotely. This corresponds to a non-significant difference in average treatment effect of 1% (95% CI: −0.5, 2.6) and an odds ratio of 1.04 (95% CI: 0.98, 1.11).

The results without mixed mode consultations are consistent with the main analysis (Table S4).

## Discussion

Using data from almost 46,000 GP consultations for ARIs in general practice in England we estimated differences in antibiotic prescribing between remote and face-to-face ARI consultations. After adjusting for a variety of factors, we found that an adult who had a remote consultation was 23% more likely to be prescribed antibiotics compared to if they had had a face-to-face consultation. In contrast, we found no evidence of an association between consultation mode and antibiotic prescribing in children.

The overall antibiotic prescribing rates by infection type are broadly similar to previously published estimates.^25^^,^^26^ However, this study adds new insight on the use of remote consultation for different infection sub-groups. Remote consultations are used for all types of ARIs, but there is variation in the proportion of remote consultations by infection type. Patients are most likely to be seen remotely for sinusitis, and least likely to be seen remotely for otitis media. As the need for antibiotics also differs by infection type, the unadjusted baseline prescribing rate for remote and face-to-face consultations are different. This shows the importance of interpreting unadjusted estimates cautiously.

Our finding that antibiotics are more likely to be prescribed to adults in remote rather than face to face consultations represents an important contribution to the field. The existing evidence from observational studies on remote consultations and antibiotic prescribing is mixed^9^, ^10^, ^11^, ^12^ but our result is consistent with survey evidence showing 67% of UK GPs believe that telehealth has increased their antibiotic prescribing.^4^ We applied TMLE to a large patient-level dataset with a wide range of variables including the infection type. The rich data means that we can control for more demographic, socioeconomic and clinical variables than most studies and the machine learning methods allow for more complex non-linear relationships between variables to be accounted for. Therefore, this provides the strongest evidence yet that antibiotic prescribing is higher in remote ARI consultations compared to face-to-face for adults.

We do not observe a difference by consultation mode in antibiotic prescribing for children. This could mean that there is no difference or that we were unable to detect it. Previous studies have found a difference in children but in different settings.^15^ The decision to prescribe depends on many factors including comorbidities and acuity. As children often have few comorbidities, acuity and other unobserved factors would be more important when prescribing antibiotics to children so there is more potential for unobserved confounding.

Including both adults and children in the same study provides us with a unique opportunity to compare results from the same practices during the same time period. A potential explanation for why we see a difference in adults but not in children could be that GPs are more risk averse when consulting with children and prefer to bring them in for a face-to-face consultation before prescribing. We do observe a higher proportion of both face-to-face and mixed consultations in children.

The factors affecting antibiotic prescribing for ARIs, and the interaction with consultation mode are complex and will require further research to disentangle. Both patients and GPs might behave differently in a face-to-face compared with a remote consultation. Total triage (patients are remotely assessed before booking into a consultation) should ensure that patients have the right type of consultation for their concern, but this system is not used by all practices, and is not perfect, especially when there is high demand for appointments. Patients may exert particular pressure on GPs in certain types of appointments. Clinical examinations, which may help to determine the need for antibiotics—such as listening to a chest or looking in an ear—are not possible in a remote consultation, which may influence prescribing. Increased GP workload has also been associated with increased prescribing of broad-spectrum antibiotics^27^ and consultations for ARIs where antibiotics are prescribed have been found to be shorter, which could also influence prescribing.^28^

There are implications for both antibiotic prescribing and the use of remote consultations. Increased prescribing in adults could have a substantial impact on the UK's commitment to reduce antibiotic prescribing by 15% by 2024, given ∼70% of antibiotics prescribing happens in primary care and that ARIs are the most common condition that antibiotics are prescribed for.^1^^,^^29^ Antibiotic prescribing declined from 2015 to 2019 but data from the pandemic is harder to interpret.^30^ Both patients and health care professionals have an important role to play in ensuring sustainable use of antibiotics. Clinical guidelines should be adapted to make sure antibiotic prescribing advice for GPs factors in remote consultations. For example, many clinical risk scores used to guide antibiotic prescribing were developed for use in face-to-face consultations, so it may be necessary for separate risk scores to be developed for use in remote consultations. It is also important to consider that remote consultations have the positive externality of not requiring unwell patients to travel to the GP surgery thereby reducing the spread of respiratory infections.

There should be continued focus on educating the public on the importance of responsible use of antibiotics. Some antimicrobial stewardship activities such as the Treat Antibiotics Responsibly, Guidance, Education and Tools initiative have been adapted to work better for both prescribers and patients in remote consultations.^1^

The risks and benefits of remote consultations in general practice are not fully understood. Further research is required to understand differences in quality of care and outcomes between remote and face-to-face consultations across a range of clinical scenarios. This study raises a concern that antibiotic prescribing rates for adults are significantly higher in remote consultations, but we do not know whether this is clinically appropriate. More work is needed to explore the appropriateness of antibiotic prescriptions across consultation modes and in different clinical contexts. That work may in turn inform our approaches to triage, by aiding understanding of clinical indications for directing patients to one consultation type over another. More research is required to investigate if any difference in antibiotic prescribing rates based on consultation mode varies across demographic groups (for example, by sex/gender or socioeconomic status).

Further quantitative analysis is needed to explore whether our findings hold true for other prescribers. This is especially important given the rapid rise in numbers of nurse practitioners, pharmacists and paramedics working in general practice. Qualitative investigation of the issue, including speaking to clinicians and patients, and observing ARI consultations to explore how antibiotic prescribing plays out in practice in remote versus face-to-face environments is also needed.

We have used a large patient-level dataset that is representative of the English population. We controlled for patient-, clinician- and practice-level factors known to be associated with antibiotic prescribing using a doubly-robust causal machine learning method. In addition, the way we classified consultations into remote, mixed and face-to-face and then grouped mixed and face-to-face consultations together, more accurately reflects the way consultations take place in general practice in England rather than using only face-to-face consultations as the comparator.

Although we included a wide range of factors, there may still be unobserved confounding, such as urgency, acuity, or staffing levels, that could influence both consultation mode and antibiotic prescribing, but we are unable to measure them in this study. This could impact the results but it is unlikely to fully remove the relationship we observe between consultation mode and antibiotic prescribing in adults.

The practices that participate in CPRD Aurum could be different to those that do not. We further limited inclusion in the study to only practices that have consented to take part in further data linkage (IMD and urban rurural index in this study). At patient level, only patients that had valid postcode recorded were included as this was necessary for the linkage. Patients in CPRD Aurum have been shown to be representative of the broader English population but it is not possible to check to what extent, if at all, the exclusion criteria applied at the practice and patient level would change this.

We are unlikely to capture all ARIs due to poor clinical coding–some studies found that only 69% of antibiotics prescribed could be linked to a specific part of the body and/or clinical condition.^31^ A further limitation of the ARI coding is that there is no distinction between primary and secondary diagnoses in CPRD Aurum. There could be a difference in the accuracy and completeness of coding (both of symptoms and antibiotic prescribing) as well as frequency of primary versus secondary diagnoses between remote and face-to-face consultations. This could lead to bias if poor coding was applied to a greater extent to either consultation mode. We only included ARI GP consultations, not other potential prescribers, and we did not differentiate between acute and delayed prescriptions or whether the prescriptions were guideline compliant.

Our results are concerning because of the potential implications for antibiotic consumption and resistance. Further research is needed to understand the causes of increased antibiotic prescribing in remote consultations, and to determine whether the observed increase is appropriate.

On a broader level, the effect of increased remote consulting in general practice needs to be considered in the round. While the rapid introduction of remote consultations was a great success in the sense that it allowed general practice to keep functioning in the first few months of the pandemic, there may be unintended consequences that follow. Careful and thorough evaluation of interventions takes on an even greater importance when changes are instituted rapidly, as was the case with the accelerated rollout of remote consultations.

## Contributors

EV and GC conceived the study. EV and KDC developed the study design and analytical approach, in consultation with other project team members. EC, EV and KDC accessed and verified the underlying data. EC processed the data sources. EV and KDC performed the statistical analyses. EV, KDC and PC drafted the initial version of the manuscript. All authors contributed to the interpretation of the findings and reviewed and edited the manuscript for intellectual content. All authors approved the final version of the manuscript and agreed to be accountable for all aspects of the work. EV is the guarantor. The corresponding author attests that all listed authors meet authorship criteria and no others meeting criteria have been omitted.

## Data sharing statement

We used deidentified primary care data from the Clinical Practice Research Datalink (CPRD). For more information, please visit: https://www.cprd.com/Data-access, and enquiries can be emailed to enquiries@cprd.gov.uk. Scientific approval for this study was given by the CPRD Independent Scientific Advisory Committee (ISAC). The primary care data can be requested via application to the Clinical Practice Research Datalink. The following link to codelists can be used to identify ARIs:https://github.com/THF-evaluative-analytics/antibiotic-prescribing-cprd/tree/main/Codelists.

CPRD has ethics approval from the Health Research Authority to support research using anonymised patient data. Requests by researchers to access the data are reviewed via the CPRD Research Data Governance (RDG) and the protocol number for this study is: 21_000357. The RDG process is to ensure that the proposed research is of benefit to patients and public health. The data used in this study were obtained from practices that had consented to participate in CPRD research, and all data were handled in accordance with CPRD guidelines for data management and confidentiality.

## Declaration of interests

We declare no competing interests.

## Figures and Tables

**combined plots remote p by infection type:**
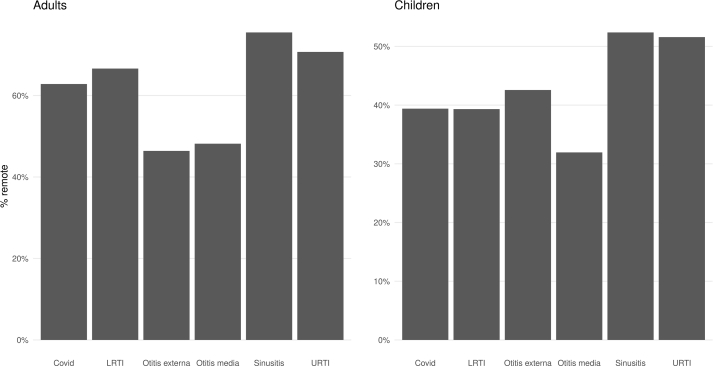


## References

[1] UK Health Security Agency (2022). https://www.gov.uk/government/publications/english-surveillance-programme-antimicrobial-utilisation-and-resistance-espaur-report.

[2] Smieszek T., Pouwels K.B., Dolk F.C.K. (2018). Potential for reducing inappropriate antibiotic prescribing in English primary care. J Antimicrob Chemother.

[3] Vestesson E.M., De Corte K.L.A., Crellin E., Ledger J., Bakhai M., Clarke G.M. (2023). Consultation rate and mode by deprivation in English general practice from 2018 to 2022: population-based study. JMIR Public Health Surveill.

[4] Beech J., Fraser C., Gardner T., Buzelli L., Williamson S., Alderwick H. (2023).

[5] Rosen R., Wieringa S., Greenhalgh T. (2022). Clinical risk in remote consultations in general practice: findings from in-COVID-19 pandemic qualitative research. BJGP Open.

[6] Gulliford M.C., Dregan A., Moore M.V. (2014). Continued high rates of antibiotic prescribing to adults with respiratory tract infection: survey of 568 UK general practices. BMJ Open.

[7] van der Velden A., Duerden M.G., Bell J. (2013). Prescriber and patient responsibilities in treatment of acute respiratory tract infections - essential for conservation of antibiotics. Antibiotics.

[8] Rose J., Crosbie M., Stewart A. (2021). https://journals.sagepub.com/doi/10.1177/1757913919879183?url_ver=Z39.88-2003&rfr_id=ori%3Arid%3Acrossref.org&rfr_dat=cr_pub++0pubmed.

[9] Shi Z., Mehrotra A., Gidengil C.A., Poon S.J., Uscher-Pines L., Ray K.N. (2018). Quality of care for acute respiratory infections during direct-to-consumer telemedicine visits for adults. Health Aff.

[10] Ray K.N., Martin J.M., Wolfson D. (2021). Antibiotic prescribing for acute respiratory tract infections during telemedicine visits within a pediatric primary care network. Acad Pediatr.

[11] Halpren-Ruder D., Chang A.M., Hollander J.E., Shah A. (2019). Quality assurance in telehealth: adherence to evidence-based indicators. Telemed J E Health.

[12] Gordon A.S., Adamson W.C., DeVries A.R. (2017). Virtual visits for acute, nonurgent care: a claims analysis of episode-level utilization. J Med Internet Res.

[13] Bakhit M., Baillie E., Krzyzaniak N. (2021). Antibiotic prescribing for acute infections in synchronous telehealth consultations: a systematic review and meta-analysis. BJGP Open.

[14] Han S.M., Greenfield G., Majeed A., Hayhoe B. (2020). http://www.jmir.org/2020/11/e23482/.

[15] Ray K.N., Shi Z., Gidengil C.A., Poon S.J., Uscher-Pines L., Mehrotra A. (2019). Antibiotic prescribing during pediatric direct-to-consumer telemedicine visits. Pediatrics.

[16] Palin V., Mölter A., Belmonte M. (2019). Antibiotic prescribing for common infections in UK general practice: variability and drivers. J Antimicrob Chemother.

[17] Hope E.C., Crump R.E., Hollingsworth T.D., Smieszek T., Robotham J.V., Pouwels K.B. (2018). Identifying English practices that are high antibiotic prescribers accounting for comorbidities and other legitimate medical reasons for variation. eClinicalMedicine.

[18] Stuart B., Brotherwood H., Van’T Hoff C. (2020). Exploring the appropriateness of antibiotic prescribing for common respiratory tract infections in UK primary care. J Antimicrob Chemother.

[19] Pouwels K.B., Dolk F.C.K., Smith D.R.M., Smieszek T., Robotham J.V. (2018). Explaining variation in antibiotic prescribing between general practices in the UK. J Antimicrob Chemother.

[20] McKay R., Patrick D.M., McGrail K., Law M.R. (2019). Antibiotic prescribing for pediatric respiratory infections: what explains a large variation among physicians?. Can Fam Physician.

[21] Thomson K., Berry R., Robinson T., Brown H., Bambra C., Todd A. (2020). An examination of trends in antibiotic prescribing in primary care and the association with area-level deprivation in England. BMC Publ Health.

[22] Mölter A., Belmonte M., Palin V. (2018). Antibiotic prescribing patterns in general medical practices in England: does area matter?. Health Place.

[23] Curtis H.J., Walker A.J., Mahtani K.R., Goldacre B. (2019). Time trends and geographical variation in prescribing of antibiotics in England 1998-2017. J Antimicrob Chemother.

[24] Borek A.J., van Hecke O., Butler C.C., Tonkin-Crine S., Pouwels K.B., Robotham J.V. (2022). Role of locum GPs in antibiotic prescribing and stewardship: a mixed-methods study. Br J Gen Pract.

[25] Nowakowska M., van Staa T., Mölter A. (2019). Antibiotic choice in UK general practice: rates and drivers of potentially inappropriate antibiotic prescribing. J Antimicrob Chemother.

[26] Pouwels K.B., Dolk F.C.K., Smith D.R.M., Robotham J v, Smieszek T. (2018). Actual versus “ideal” antibiotic prescribing for common conditions in English primary care. J Antimicrob Chemother.

[27] Allen T., Gyrd-Hansen D., Kristensen S.R., Oxholm A.S., Pedersen L.B., Pezzino M. (2022). Physicians under pressure: evidence from antibiotics prescribing in England. Med Decis Making.

[28] Martinez K.A., Rood M., Jhangiani N., Kou L., Boissy A., Rothberg M.B. (2018). Association between antibiotic prescribing for respiratory tract infections and patient satisfaction in direct-to-consumer telemedicine. JAMA Intern Med.

[29] HM Government (2019). https://www.gov.uk/government/publications/uk-5-year-action-plan-for-antimicrobial-resistance-2019-to-2024https://www.gov.uk/government/publications/uk-5-year-action-plan-for-antimicrobial-resistance-2019-to-2024.

[30] ESPAUR (2020). http://www.gov.uk/phe%5Cnwww.facebook.com/PublicHealthEngland.

[31] Dolk F.C.K., Pouwels K.B., Smith D.R.M., Robotham J.V., Smieszek T. (2018). Antibiotics in primary care in England: which antibiotics are prescribed and for which conditions?. J Antimicrob Chemother.

