# Supplementary material for: Antibiotic prescribing in remote versus face-to-face consultations for acute respiratory infections in primary care in England: an observational study using target maximum likelihood estimation
Source: eClinicalMedicine. 2023 Oct 3;64:102245. doi: 10.1016/j.eclinm.2023.102245 (PMC10568332; doi:10.1016/j.eclinm.2023.102245)
Supplement: Supplementary Material [file mmc1.docx]

Supplemental materials

Supplementary materials

S1. Link to codelists to identify ARIs.

<https://github.com/THF-evaluative-analytics/antibiotic-prescribing-cprd/tree/main/Codelists>

Table with codelists

| list_name | code | description | medcodeid |
| --- | --- | --- | --- |
| 745 - res70: Cough/cold | 16L..00 | Influenza-like symptoms | 460161011 |
| 745 - res70: Cough/cold | 171..00 | Cough | 82824016 |
| 745 - res70: Cough/cold | 171..11 | C/O - cough | 407081018 |
| 745 - res70: Cough/cold | 1712 | Dry cough | 20419011 |
| 745 - res70: Cough/cold | 1713 | Productive cough -clear sputum | 252351017 |
| 745 - res70: Cough/cold | 1714 | Productive cough -green sputum | 252352012 |
| 745 - res70: Cough/cold | 1715 | Productive cough-yellow sputum | 252353019 |
| 745 - res70: Cough/cold | 1716 | Productive cough NOS | 397882011 |
| 745 - res70: Cough/cold | 1716.11 | Coughing up phlegm | 598431000000000 |
| 745 - res70: Cough/cold | 1719 | Chesty cough | 252359015 |
| 745 - res70: Cough/cold | 1719.11 | Bronchial cough | 252360013 |
| 745 - res70: Cough/cold | 171B.00 | Persistent cough | 423230012 |
| 745 - res70: Cough/cold | 171F.00 | Cough with fever | 216653013 |
| 745 - res70: Cough/cold | 171Z.00 | Cough symptom NOS | 252366019 |
| 745 - res70: Cough/cold | A33..00 | Whooping cough | 46589015 |
| 745 - res70: Cough/cold | H00..00 | Acute nasopharyngitis | 136470019 |
| 745 - res70: Cough/cold | H00..11 | Common cold | 136463019 |
| 745 - res70: Cough/cold | H00..12 | Coryza - acute | 598191000000000 |
| 745 - res70: Cough/cold | H00..13 | Febrile cold | 667611000000000 |
| 745 - res70: Cough/cold | H00..14 | Nasal catarrh - acute | 681561000000000 |
| 745 - res70: Cough/cold | H00..15 | Pyrexial cold | 195731000000000 |
| 745 - res70: Cough/cold | H00..16 | Rhinitis - acute | 162421000000000 |
| 745 - res70: Cough/cold | H07..00 | Chest cold | 301143015 |
| 745 - res70: Cough/cold | H27z.11 | Flu like illness | 762181000000000 |
| 745 - res70: Cough/cold | H27z.12 | Influenza like illness | 778711000000000 |
| 745 - res70: Cough/cold | R062.00 | [D]Cough | 317403015 |
| 745 - res70: Cough/cold | R2...13 | cold | 303571000000000 |
| 736 - res70: LRTI | H06..00 | Acute bronchitis and bronchiolitis | 301095014 |
| 736 - res70: LRTI | H060.00 | Acute bronchitis | 18268014 |
| 736 - res70: LRTI | H060000 | Acute fibrinous bronchitis | 301100011 |
| 736 - res70: LRTI | H060.11 | Acute wheezy bronchitis | 411490016 |
| 736 - res70: LRTI | H060300 | Acute purulent bronchitis | 301103013 |
| 736 - res70: LRTI | H060400 | Acute croupous bronchitis | 2475602011 |
| 736 - res70: LRTI | H060500 | Acute tracheobronchitis | 58909014 |
| 736 - res70: LRTI | H060600 | Acute pneumococcal bronchitis | 301105018 |
| 736 - res70: LRTI | H060700 | Acute streptococcal bronchitis | 301106017 |
| 736 - res70: LRTI | H060800 | Acute haemophilus influenzae bronchitis | 301108016 |
| 736 - res70: LRTI | H060A00 | Acute bronchitis due to mycoplasma pneumoniae | 350044014 |
| 736 - res70: LRTI | H060x00 | Acute bacterial bronchitis unspecified | 301121011 |
| 736 - res70: LRTI | H060z00 | Acute bronchitis NOS | 301122016 |
| 736 - res70: LRTI | H061.00 | Acute bronchiolitis | 10187013 |
| 736 - res70: LRTI | H061000 | Acute capillary bronchiolitis | 2478755014 |
| 736 - res70: LRTI | H061200 | Acute bronchiolitis with bronchospasm | 25801011 |
| 736 - res70: LRTI | H061300 | Acute exudative bronchiolitis | 301126018 |
| 736 - res70: LRTI | H061z00 | Acute bronchiolitis NOS | 301130015 |
| 736 - res70: LRTI | H062.00 | Acute lower respiratory tract infection | 457801000000000 |
| 736 - res70: LRTI | H06z.00 | Acute bronchitis or bronchiolitis NOS | 301132011 |
| 736 - res70: LRTI | H06z000 | Chest infection NOS | 396090018 |
| 736 - res70: LRTI | H06z011 | Chest infection | 546411000000000 |
| 736 - res70: LRTI | H06z100 | Lower resp tract infection | 733471000000000 |
| 736 - res70: LRTI | H06z111 | Respiratory tract infection | 411488017 |
| 736 - res70: LRTI | H06z112 | Acute lower respiratory tract infection | 579878017 |
| 736 - res70: LRTI | H06z200 | Recurrent chest infection | 451444011 |
| 736 - res70: LRTI | H24..11 | Chest infection with infectious disease EC | 546511000000000 |
| 736 - res70: LRTI | H30..00 | Bronchitis unspecified | 396107011 |
| 736 - res70: LRTI | H300.00 | Tracheobronchitis NOS | 301437010 |
| 736 - res70: LRTI | H301.00 | Laryngotracheobronchitis | 142425010 |
| 736 - res70: LRTI | H30..11 | Chest infection - unspecified bronchitis | 350041018 |
| 736 - res70: LRTI | H30..12 | Recurrent wheezy bronchitis | 183191000000000 |
| 736 - res70: LRTI | H302.00 | Wheezy bronchitis | 2163183015 |
| 736 - res70: LRTI | H30z.00 | Bronchitis NOS | 301441014 |
| 736 - res70: LRTI | H34..00 | Bronchiectasis | 21163015 |
| 736 - res70: LRTI | H340.00 | Recurrent bronchiectasis | 301524014 |
| 736 - res70: LRTI | H341.00 | Post-infective bronchiectasis | 301525010 |
| 736 - res70: LRTI | H34z.00 | Bronchiectasis NOS | 301526011 |
| 736 - res70: LRTI | H50..00 | Empyema | 456436010 |
| 736 - res70: LRTI | H500100 | Empyema with bronchopleural fistula | 47025013 |
| 736 - res70: LRTI | H501200 | Pleural empyema | 218821000000000 |
| 736 - res70: LRTI | H501400 | Purulent pleurisy | 1231843013 |
| 736 - res70: LRTI | H51..00 | Pleurisy | 301644013 |
| 736 - res70: LRTI | H510300 | Acute dry pleurisy | 301647018 |
| 736 - res70: LRTI | H510900 | Pneumococcal pleurisy | 5407016 |
| 736 - res70: LRTI | H583200 | Eosinophilic bronchitis | 2240630000000000 |
| 739 - res70: Otitis externa | 2D95.00 | O/E - tympanic membrane red | 255652011 |
| 739 - res70: Otitis externa | F500.00 | Perichondritis of pinna | 56994016 |
| 739 - res70: Otitis externa | F500000 | Unspecified perichondritis of pinna | 298935015 |
| 739 - res70: Otitis externa | F500100 | Acute perichondritis of pinna | 76474017 |
| 739 - res70: Otitis externa | F500200 | Chronic pinna perichondritis | 219451000000000 |
| 739 - res70: Otitis externa | F500211 | Chondrodermatitis nodularis helicis | 552351000000000 |
| 739 - res70: Otitis externa | F500300 | Chondrodermatitis nodularis helicis | 127176015 |
| 739 - res70: Otitis externa | F500z00 | Perichondritis of pinna NOS | 298942015 |
| 739 - res70: Otitis externa | F501.00 | Infective otitis externa | 144257010 |
| 739 - res70: Otitis externa | F501000 | Unspecified infective otitis externa | 298945018 |
| 739 - res70: Otitis externa | F501100 | Acute infective otitis externa | 399494015 |
| 739 - res70: Otitis externa | F501111 | Abscess; external ear | 219461000000000 |
| 739 - res70: Otitis externa | F501112 | Cellulitis; external ear | 219471000000000 |
| 739 - res70: Otitis externa | F501200 | Acute infection of pinna | 94233010 |
| 739 - res70: Otitis externa | F501300 | Acute swimmers' ear | 460581000000000 |
| 739 - res70: Otitis externa | F501400 | Infective otitis externa due to erysipelas | 298954015 |
| 739 - res70: Otitis externa | F501411 | Erysipelas - otitis externa | 298955019 |
| 739 - res70: Otitis externa | F501700 | Infective otitis externa due to impetigo | 298959013 |
| 739 - res70: Otitis externa | F501711 | Impetigo - otitis externa | 298960015 |
| 739 - res70: Otitis externa | F501900 | Other acute external ear infections | 298964012 |
| 739 - res70: Otitis externa | F501E00 | Other chronic infective otitis externa | 298969019 |
| 739 - res70: Otitis externa | F501F00 | Chronic infective otitis externa NOS | 298970018 |
| 739 - res70: Otitis externa | F501G00 | Haemorrhagic otitis externa | 56652016 |
| 739 - res70: Otitis externa | F501y00 | Other specified infective otitis externa | 298973016 |
| 739 - res70: Otitis externa | F501z00 | Infective otitis externa NOS | 298974010 |
| 739 - res70: Otitis externa | F502.00 | Other otitis externa | 298975011 |
| 739 - res70: Otitis externa | F502z00 | Otitis externa NOS | 399496018 |
| 739 - res70: Otitis externa | F502z11 | Inflammation ear external | 779301000000000 |
| 739 - res70: Otitis externa | F506.00 | Abscess of external ear | 347926013 |
| 739 - res70: Otitis externa | F586.00 | Otorrhoea | 109102019 |
| 739 - res70: Otitis externa | F586000 | Unspecified otorrhoea | 399506010 |
| 739 - res70: Otitis externa | F586011 | Discharging ear NOS | 622921000000000 |
| 739 - res70: Otitis externa | F586200 | Otorrhagia | 44084019 |
| 739 - res70: Otitis externa | F586z00 | Otorrhoea NOS | 299229016 |
| 739 - res70: Otitis externa | F587.00 | Otalgia | 27089013 |
| 739 - res70: Otitis externa | F587000 | Unspecified otalgia | 299233011 |
| 739 - res70: Otitis externa | F587.11 | Ear pain | 477607011 |
| 739 - res70: Otitis externa | F587z00 | Otalgia NOS | 299234017 |
| 740 - res70: Otitis media | A552.00 | Postmeasles otitis media | 22934019 |
| 740 - res70: Otitis media | F51..00 | Nonsuppurative otitis media + eustachian tube disorders | 297571000000000 |
| 740 - res70: Otitis media | F510.00 | Acute non suppurative otitis media | 458531000000000 |
| 740 - res70: Otitis media | F510000 | Acute otitis media with effusion | 58707018 |
| 740 - res70: Otitis media | F510011 | Acute secretory otitis media | 473798017 |
| 740 - res70: Otitis media | F510100 | Acute serous otitis media | 299014013 |
| 740 - res70: Otitis media | F510200 | Acute mucoid otitis media | 87124011 |
| 740 - res70: Otitis media | F510300 | Acute sanguinous otitis media | 128622017 |
| 740 - res70: Otitis media | F510z00 | Acute nonsuppurative otitis media NOS | 458581000000000 |
| 740 - res70: Otitis media | F511.00 | Chronic otitis media with effusion; serous | 555791000000000 |
| 740 - res70: Otitis media | F511.11 | Chronic secretory otitis media; serous | 504727012 |
| 740 - res70: Otitis media | F511z00 | Chronic serous otitis media NOS | 299024017 |
| 740 - res70: Otitis media | F512.00 | Chronic otitis media with effusion; mucoid | 555761000000000 |
| 740 - res70: Otitis media | F512000 | Glue ear; unspecified | 299030017 |
| 740 - res70: Otitis media | F512.11 | Glue ear | 130848015 |
| 740 - res70: Otitis media | F512.12 | Chronic secretory otitis media; mucoid | 503932010 |
| 740 - res70: Otitis media | F512z00 | Chronic mucoid otitis media NOS | 299032013 |
| 740 - res70: Otitis media | F513.00 | Chronic otitis media with effusion; other | 299034014 |
| 740 - res70: Otitis media | F513100 | Chronic otitis media with effusion; purulent | 490746013 |
| 740 - res70: Otitis media | F513111 | Chronic secretory otitis media; purulent | 490749018 |
| 740 - res70: Otitis media | F514.00 | Unspecified nonsuppurative otitis media | 77591000000000 |
| 740 - res70: Otitis media | F514100 | Serous otitis media NOS | 299040019 |
| 740 - res70: Otitis media | F514200 | Catarrhal otitis media NOS | 299041015 |
| 740 - res70: Otitis media | F514300 | Mucoid otitis media NOS | 299042010 |
| 740 - res70: Otitis media | F514z00 | Nonsuppurative otitis media NOS | 297581000000000 |
| 740 - res70: Otitis media | F515.00 | Eustachian tube salpingitis | 405110011 |
| 740 - res70: Otitis media | F515.11 | Catarrh - eustachian | 399840017 |
| 740 - res70: Otitis media | F52..00 | Suppurative and unspecified otitis media | 116801000000000 |
| 740 - res70: Otitis media | F520.00 | Acute suppurative otitis media | 299061014 |
| 740 - res70: Otitis media | F520000 | Acute suppurative otitis media tympanic membrane intact | 460541000000000 |
| 740 - res70: Otitis media | F520100 | Acute suppurative otitis media tympanic membrane ruptured | 460551000000000 |
| 740 - res70: Otitis media | F520300 | Acute suppurative otitis media due to disease EC | 299065017 |
| 740 - res70: Otitis media | F520z00 | Acute suppurative otitis media NOS | 299066016 |
| 740 - res70: Otitis media | F521.00 | Chronic suppurative otitis media; tubotympanic | 556821000000000 |
| 740 - res70: Otitis media | F522.00 | Chronic suppurative otitis media; atticoantral | 556811000000000 |
| 740 - res70: Otitis media | F523.00 | Chronic suppurative otitis media NOS | 299067013 |
| 740 - res70: Otitis media | F524.00 | Purulent otitis media NOS | 299068015 |
| 740 - res70: Otitis media | F524000 | Bilateral suppurative otitis media | 299069011 |
| 740 - res70: Otitis media | F525.00 | Recurrent acute otitis media | 299070012 |
| 740 - res70: Otitis media | F526.00 | Acute left otitis media | 299071011 |
| 740 - res70: Otitis media | F527.00 | Acute right otitis media | 299072016 |
| 740 - res70: Otitis media | F528.00 | Acute bilateral otitis media | 299073014 |
| 740 - res70: Otitis media | F52z.00 | Otitis media NOS | 399498017 |
| 740 - res70: Otitis media | F52z.11 | Infection ear | 780361000000000 |
| 740 - res70: Otitis media | SN30.11 | Aero-otitis media | 82051019 |
| 746 - res70: Quinsy | H15..00 | Peritonsillar abscess - quinsy | 229081000000000 |
| 746 - res70: Quinsy | H15..11 | Quinsy | 25522012 |
| 741 - res70: Sinusitis | H01..00 | Acute sinusitis | 26785019 |
| 741 - res70: Sinusitis | H010.00 | Acute maxillary sinusitis | 113403018 |
| 741 - res70: Sinusitis | H011.00 | Acute frontal sinusitis | 150861013 |
| 741 - res70: Sinusitis | H01..11 | Sinusitis | 61668014 |
| 741 - res70: Sinusitis | H012.00 | Acute ethmoidal sinusitis | 112674013 |
| 741 - res70: Sinusitis | H013.00 | Acute sphenoidal sinusitis | 129311019 |
| 741 - res70: Sinusitis | H014.00 | Acute rhinosinusitis | 567061000000000 |
| 741 - res70: Sinusitis | H01y.00 | Other acute sinusitis | 301013015 |
| 741 - res70: Sinusitis | H01yz00 | Other acute sinusitis NOS | 301014014 |
| 741 - res70: Sinusitis | H01z.00 | Acute sinusitis NOS | 301015010 |
| 741 - res70: Sinusitis | H13..00 | Chronic sinusitis | 66718013 |
| 741 - res70: Sinusitis | H130.00 | Chronic maxillary sinusitis | 59944016 |
| 741 - res70: Sinusitis | H130.11 | Antritis - chronic | 488111000000000 |
| 741 - res70: Sinusitis | H130.12 | Maxillary sinusitis | 146480018 |
| 741 - res70: Sinusitis | H131.00 | Chronic frontal sinusitis | 99894013 |
| 741 - res70: Sinusitis | H13..11 | Chronic rhinosinusitis | 1229598015 |
| 741 - res70: Sinusitis | H131.11 | Frontal sinusitis | 130650014 |
| 741 - res70: Sinusitis | H132.00 | Chronic ethmoidal sinusitis | 121629017 |
| 741 - res70: Sinusitis | H134.00 | Fistula of nasal sinus | 301209018 |
| 741 - res70: Sinusitis | H135.00 | Recurrent sinusitis | 301210011 |
| 741 - res70: Sinusitis | H13y.00 | Other chronic sinusitis | 301211010 |
| 741 - res70: Sinusitis | H13y000 | Chronic pansinusitis | 147320013 |
| 741 - res70: Sinusitis | H13y100 | Pansinusitis | 301212015 |
| 741 - res70: Sinusitis | H13yz00 | Other chronic sinusitis NOS | 301213013 |
| 741 - res70: Sinusitis | H13z.00 | Chronic sinusitis NOS | 301214019 |
| 747 - res70: Sore throat | 1C9..00 | Sore throat symptom | 398001015 |
| 747 - res70: Sore throat | 1C9..11 | Throat soreness | 2164212016 |
| 747 - res70: Sore throat | 1C9Z.00 | Sore throat symptom NOS | 253227017 |
| 747 - res70: Sore throat | AA1..00 | Vincent's angina | 2470021018 |
| 747 - res70: Sore throat | AA10.00 | Vincent's stomatitis | 268763018 |
| 747 - res70: Sore throat | AA11.00 | Vincent's gingivitis | 267378012 |
| 747 - res70: Sore throat | AA1..11 | Trench mouth | 1786648011 |
| 747 - res70: Sore throat | AA12.00 | Vincent's pharyngitis | 60991000000000 |
| 747 - res70: Sore throat | AA1z.00 | Vincent's angina NOS | 395416013 |
| 747 - res70: Sore throat | AA1z.11 | Vincent's laryngitis | 348220011 |
| 747 - res70: Sore throat | AA1z.12 | Vincent's tonsillitis | 348206016 |
| 747 - res70: Sore throat | H02..00 | Acute pharyngitis | 486416017 |
| 747 - res70: Sore throat | H021.00 | Acute phlegmonous pharyngitis | 301022019 |
| 747 - res70: Sore throat | H02..11 | Sore throat NOS | 139761000000000 |
| 747 - res70: Sore throat | H02..12 | Viral sore throat NOS | 61411000000000 |
| 747 - res70: Sore throat | H02..13 | Throat infection - pharyngitis | 100101000000000 |
| 747 - res70: Sore throat | H022.00 | Acute ulcerative pharyngitis | 301023012 |
| 747 - res70: Sore throat | H023.00 | Acute bacterial pharyngitis | 301024018 |
| 747 - res70: Sore throat | H023z00 | Acute bacterial pharyngitis NOS | 301027013 |
| 747 - res70: Sore throat | H02z.00 | Acute pharyngitis NOS | 459581000000000 |
| 747 - res70: Sore throat | H03..00 | Acute tonsillitis | 29982014 |
| 747 - res70: Sore throat | H030.00 | Acute erythematous tonsillitis | 301035011 |
| 747 - res70: Sore throat | H031.00 | Acute follicular tonsillitis | 301036012 |
| 747 - res70: Sore throat | H03..11 | Throat infection - tonsillitis | 100111000000000 |
| 747 - res70: Sore throat | H03..12 | Tonsillitis | 149482010 |
| 747 - res70: Sore throat | H032.00 | Acute ulcerative tonsillitis | 301037015 |
| 747 - res70: Sore throat | H033.00 | Acute catarrhal tonsillitis | 301038013 |
| 747 - res70: Sore throat | H035.00 | Acute bacterial tonsillitis | 301041016 |
| 747 - res70: Sore throat | H035100 | Acute staphylococcal tonsillitis | 301043018 |
| 747 - res70: Sore throat | H035z00 | Acute bacterial tonsillitis NOS | 301044012 |
| 747 - res70: Sore throat | H037.00 | Recurrent acute tonsillitis | 301047017 |
| 747 - res70: Sore throat | H03z.00 | Acute tonsillitis NOS | 301049019 |
| 747 - res70: Sore throat | H14y500 | Caseous tonsillitis | 301234018 |
| 747 - res70: Sore throat | H14y600 | Lingular tonsillitis | 301235017 |
| 747 - res70: Sore throat | R041.00 | [D]Throat pain | 317269017 |
| 749 - res70: Sore throat_bacterial | A34..00 | Streptococcal sore throat and scarlatina | 121591000000000 |
| 749 - res70: Sore throat_bacterial | A340.00 | Streptococcal sore throat | 73157016 |
| 749 - res70: Sore throat_bacterial | A340000 | Streptococcal angina | 73160011 |
| 749 - res70: Sore throat_bacterial | A340100 | Streptococcal laryngitis | 141055011 |
| 749 - res70: Sore throat_bacterial | A340200 | Streptococcal pharyngitis | 73158014 |
| 749 - res70: Sore throat_bacterial | A340300 | Streptococcal tonsillitis | 69365016 |
| 749 - res70: Sore throat_bacterial | A340z00 | Streptococcal sore throat NOS | 286457014 |
| 749 - res70: Sore throat_bacterial | A341.00 | Scarlet fever - scarlatina | 154821000000000 |
| 749 - res70: Sore throat_bacterial | A341.11 | Scarlet fever | 50618013 |
| 749 - res70: Sore throat_bacterial | A341.12 | Scarlatina | 50619017 |
| 749 - res70: Sore throat_bacterial | A34z.00 | Streptococcal sore throat with scarlatina NOS | 286458016 |
| 749 - res70: Sore throat_bacterial | J083.00 | Oral cellulitis and abscess | 42341000000000 |
| 749 - res70: Sore throat_bacterial | J083600 | Uvulitis | 442131015 |
| 748 - res70: URTI | H0...00 | Acute respiratory infections | 300997012 |
| 748 - res70: URTI | H04..00 | Acute laryngitis and tracheitis | 412576019 |
| 748 - res70: URTI | H040.00 | Acute laryngitis | 12028011 |
| 748 - res70: URTI | H040000 | Acute oedematous laryngitis | 301052010 |
| 748 - res70: URTI | H040100 | Acute ulcerative laryngitis | 301053017 |
| 748 - res70: URTI | H040200 | Acute catarrhal laryngitis | 301054011 |
| 748 - res70: URTI | H040300 | Acute phlegmonous laryngitis | 301055012 |
| 748 - res70: URTI | H040600 | Acute suppurative laryngitis | 301059018 |
| 748 - res70: URTI | H040x00 | Acute bacterial laryngitis unspecified | 301064019 |
| 748 - res70: URTI | H040z00 | Acute laryngitis NOS | 301065018 |
| 748 - res70: URTI | H041.00 | Acute tracheitis | 44623012 |
| 748 - res70: URTI | H041000 | Acute tracheitis without obstruction | 106996011 |
| 748 - res70: URTI | H041100 | Acute tracheitis with obstruction | 15053015 |
| 748 - res70: URTI | H041z00 | Acute tracheitis NOS | 301068016 |
| 748 - res70: URTI | H042.00 | Acute laryngotracheitis | 107009017 |
| 748 - res70: URTI | H042000 | Acute laryngotracheitis without obstruction | 63301015 |
| 748 - res70: URTI | H042100 | Acute laryngotracheitis with obstruction | 99622016 |
| 748 - res70: URTI | H042.11 | Laryngotracheitis | 91647017 |
| 748 - res70: URTI | H042z00 | Acute laryngotracheitis NOS | 301074016 |
| 748 - res70: URTI | H043.00 | Acute epiglottitis (non strep) | 456591000000000 |
| 748 - res70: URTI | H043000 | Acute epiglottitis without obstruction | 83125011 |
| 748 - res70: URTI | H043200 | Acute obstructive laryngitis | 1.00161E+015 |
| 748 - res70: URTI | H043z00 | Acute epiglottitis NOS | 301080012 |
| 748 - res70: URTI | H04z.00 | Acute laryngitis and tracheitis NOS | 301082016 |
| 748 - res70: URTI | H05..00 | Other acute upper respiratory infections | 301083014 |
| 748 - res70: URTI | H050.00 | Acute laryngopharyngitis | 92029013 |
| 748 - res70: URTI | H051.00 | Acute upper respiratory tract infection | 460751000000000 |
| 748 - res70: URTI | H052.00 | Pharyngotracheitis | 301087010 |
| 748 - res70: URTI | H053.00 | Tracheopharyngitis | 301086018 |
| 748 - res70: URTI | H054.00 | Recurrent upper respiratory tract infection | 301088017 |
| 748 - res70: URTI | H055.00 | Pharyngolaryngitis | 301089013 |
| 748 - res70: URTI | H05y.00 | Other upper respiratory infections of multiple sites | 301090016 |
| 748 - res70: URTI | H05z.00 | Upper respiratory infection NOS | 396089010 |
| 748 - res70: URTI | H05z.11 | Upper respiratory tract infection NOS | 73091000000000 |
| 748 - res70: URTI | H0y..00 | Other specified acute respiratory infections | 301144014 |
| 748 - res70: URTI | H0z..00 | Acute respiratory infection NOS | 301145010 |
| 748 - res70: URTI | H160100 | Chronic catarrhal laryngitis | 301243010 |
| 748 - res70: URTI | Hyu0.00 | [X]Acute upper respiratory infections | 362471000000000 |
| 748 - res70: URTI | Hyu0100 | [X]Acute pharyngitis due to other specified organisms | 301802017 |
| 748 - res70: URTI | Hyu0200 | [X]Acute tonsillitis due to other specified organisms | 301803010 |
| Cough/cold | 1719 | chesty cough | 252359015 |
| Cough/cold | NA | chesty cough | 1.37687610000061E+016 |
| Cough/cold | 171..00 | cough | 82824016 |
| Cough/cold | NA | cough | 961781000006119 |
| LRTI | NA | lrti - lower respiratory tract infection | 3316401000006114 |
| LRTI | NA | lower respiratory infection caused by sars-cov-2 (severe acute respiratory syndrome coronavirus 2) | 1.34867410000061E+016 |
| Otitis media | NA | acute otitis media | 1806411000006119 |
| Otitis media | NA | chronic otitis media | 2836821000006119 |
| Otitis media | F513199 | chronic purulent otitis media | 63478014 |
| Otitis media | F523.99 | chronic purulent otitis media | 883741000006115 |
| Otitis media | F513199 | chronic purulent otitis media | 63478014 |
| Otitis media | F523.99 | chronic purulent otitis media | 883741000006115 |
| Otitis media | F513199 | chronic purulent otitis media | 63478014 |
| Otitis media | F523.99 | chronic purulent otitis media | 883741000006115 |
| Otitis media | NA | chronic otitis media with perforation | 3118481000006112 |
| Sinusitis | NA | viral sinusitis | 7298311000006115 |
| Sinusitis | H13..11 | chronic rhinosinusitis | 1229598015 |
| Sinusitis | NA | chronic rhinosinusitis | 1.41498610000061E+016 |
| Sinusitis | H13..11 | chronic rhinosinusitis | 1229598015 |
| Sinusitis | NA | chronic rhinosinusitis | 1.41498610000061E+016 |
| Sore throat | NA | acute tonsilitis/pharyngitis | 855671000006111 |
| urti | NA | urti - viral upper respiratory tract infection | 5655871000006119 |
| urti | H05z.12 | viral upper respiratory tract infection | 350040017 |

S2. Table of outcome, exposure and covariates included in models

| variable | adults | children | variable type | Role in model |
| --- | --- | --- | --- | --- |
| abx_prescribed | Yes | Yes | Binary | Outcome |
| remote_cons | Yes | Yes | Binary | Exposure |
| age_cons | Yes | Yes | Continuous | Covariate |
| ethnic_group | Yes | Yes | Categorical | Covariate |
| sex | Yes | Yes | Binary | Covariate |
| urban | Yes | Yes | Binary | Covariate |
| region | Yes | Yes | Categorical | Covariate |
| abx_per_10000_pd | Yes | Yes | Continuous | Covariate |
| covid_p | Yes | Yes | Continuous | Covariate |
| cons_per_practice_per_10000_days | Yes | Yes | Continuous | Covariate |
| imd2015_5 | Yes | Yes | Categorical | Covariate |
| day_of_week | Yes | Yes | Categorical | Covariate |
| month_counter | Yes | Yes | Continuous | Covariate |
| jobcat_general_medical_practitioner | Yes | Yes | Binary | Covariate |
| jobcat_locum_gp | Yes | Yes | Binary | Covariate |
| jobcat_gp_registrar | Yes | Yes | Binary | Covariate |
| jobcat_salaried_general_practitioner | Yes | Yes | Binary | Covariate |
| jobcat_sessional_gp | Yes | Yes | Binary | Covariate |
| jobcat_associate_practitioner_general_practitioner | Yes | Yes | Binary | Covariate |
| jobcat_assistant_gp | Yes | Yes | Binary | Covariate |
| ncd_other_cancer | Yes | Yes | Binary | Covariate |
| ncd_psychosis | Yes | No | Binary | Covariate |
| ncd_dementia | Yes | No | Binary | Covariate |
| ncd_depression_medcode | Yes | Yes | Binary | Covariate |
| ncd_anxiety_medcode | Yes | No | Binary | Covariate |
| ncd_skin_condition | Yes | Yes | Binary | Covariate |
| ncd_asthma | Yes | Yes | Binary | Covariate |
| ncd_hypertension | Yes | Yes | Binary | Covariate |
| ncd_obesity | Yes | Yes | Binary | Covariate |
| ncd_other_genitourinary | Yes | Yes | Binary | Covariate |
| ncd_neurological_condition | Yes | Yes | Binary | Covariate |
| ncd_other_musculoskeletal | Yes | Yes | Binary | Covariate |
| ncd_haemm_immun_condition | Yes | Yes | Binary | Covariate |
| ncd_eye_condition | Yes | Yes | Binary | Covariate |
| ncd_epilepsy | Yes | Yes | Binary | Covariate |
| ncd_infectious_disease_hiv_viral_hep | Yes | No | Binary | Covariate |
| ncd_ear_condition | Yes | Yes | Binary | Covariate |
| ncd_copd | Yes | No | Binary | Covariate |
| ncd_other_endocrine | Yes | No | Binary | Covariate |
| ncd_other_respiratory | Yes | Yes | Binary | Covariate |
| ncd_chronic_liver_disease | Yes | No | Binary | Covariate |
| ncd_other_digestive | Yes | No | Binary | Covariate |
| ncd_other_circulatory | Yes | Yes | Binary | Covariate |
| ncd_connective_tissue_disorder | Yes | No | Binary | Covariate |
| infection_type_urti | Yes | Yes | Binary | Covariate |
| infection_type_covid | Yes | Yes | Binary | Covariate |
| infection_type_otitis_media | Yes | Yes | Binary | Covariate |
| infection_type_lrti | Yes | Yes | Binary | Covariate |
| infection_type_sinusitis | Yes | Yes | Binary | Covariate |
| infection_type_otitis_externa | Yes | Yes | Binary | Covariate |
| infection_type_count | Yes | Yes | Binary | Covariate |
| cons_30 | Yes | Yes | Continuous | Covariate |
| remote_30 | Yes | Yes | Continuous | Covariate |
| f2f_30 | Yes | Yes | Continuous | Covariate |
| abx_30 | Yes | Yes | Continuous | Covariate |
| cons_arti_30 | Yes | Yes | Continuous | Covariate |
| remote_arti_30 | Yes | Yes | Continuous | Covariate |
| f2f_arti_30 | Yes | Yes | Continuous | Covariate |
| cons_365 | Yes | Yes | Continuous | Covariate |
| remote_365 | Yes | Yes | Continuous | Covariate |
| f2f_365 | Yes | Yes | Continuous | Covariate |
| abx_365 | Yes | Yes | Continuous | Covariate |
| cons_arti_365 | Yes | Yes | Continuous | Covariate |
| remote_arti_365 | Yes | Yes | Continuous | Covariate |
| f2f_arti_365 | Yes | Yes | Continuous | Covariate |
| cons_7 | Yes | Yes | Continuous | Covariate |
| remote_7 | Yes | Yes | Continuous | Covariate |
| f2f_7 | Yes | Yes | Continuous | Covariate |
| abx_7 | Yes | Yes | Continuous | Covariate |
| cons_arti_7 | Yes | Yes | Continuous | Covariate |
| remote_arti_7 | Yes | Yes | Continuous | Covariate |
| f2f_arti_7 | Yes | Yes | Continuous | Covariate |
| cons_covid_7 | Yes | Yes | Continuous | Covariate |

S3. Algorithm selection and model tuning

The algorithm selection and the number of cross-validation folds was based on the principles outlined in “Practical considerations for specifying a super learner”. The effective sample size was calculated by multiplying the unique number of patients by the proportion of consultations that lead to an antibiotic prescription. The effective sample size was larger than 500 but less than 5,000 so 10-fold cross-validation was used and as many as learners as computationally realistic were included. There were continuous covariates so learners that do not enforce relationships to be linear or monotonic such as regression splines and random forest were included. Two different types of tree-based models, Random Forest (using bagging) and XGBoost (using boosting), were included due to their distinct strengths, which complement each other and make them well-suited for this type of setting.

Phillips R V, van der Laan MJ, Lee H, Gruber S. Practical considerations for specifying a super learner. Int J Epidemiol [Internet]. 2023 Mar 11;dyad023. Available from: https://doi.org/10.1093/ije/dyad023

For the XGBoost model, we tuned the parameters min_n, tree_depth, and learn_rate. Here, min_n specifies the minimum number of observations required in a node for a split to be considered, tree_depth controls the maximum depth of the trees, and learn_rate scales the contribution of each tree in the ensemble. We generated a grid of 2 levels for each of these parameters, leading to the specification of 8 different XGBoost models, each with a unique combination of parameters.

For the Random Forest model, the tuned parameters are min_n and tree_depth. Similar to the XGBoost model, min_n determines the minimum number of samples required to split an internal node, and tree_depth defines the maximum depth of the trees. We also used a regular grid of 2 levels for these parameters, resulting in the specification of 4 distinct Random Forest models.

S4. Outcome model excluding mixed consultations

We estimated that 44.2% (95% CI: 43.1, 45.3) of adults would have been prescribed antibiotics if they had been seen face-to-face with 50.0% (95% CI: 49.3, 50.6) if seen remotely, which corresponds to a difference in average treatment effect of 5.7% (95% CI: 4.5, 6.9) and an odds ratio of 1.26 (95% CI: 1.20, 1.32) after adjustment for a variety of demographic, clinical and socio-economic factors using TMLE.

In children, we estimated that 42.7% (95% CI: 41.2, 44.2) would have been prescribed antibiotics if they had been seen face-to-face with 43.0% (95% CI: 41.8, 44.9) if seen remotely. This corresponds to a non-significant difference in average treatment effect of 0.5% (95% CI: -1.4, 2.3) and an odds ratio of 1.01 (95% CI: 0.94, 1.09)
